# Effects of 3 Weeks of Water Immersion and Restraint Stress on Sleep in Mice

**DOI:** 10.3389/fnins.2019.01072

**Published:** 2019-10-14

**Authors:** Shinnosuke Yasugaki, Chih-Yao Liu, Mitsuaki Kashiwagi, Mika Kanuka, Takato Honda, Shingo Miyata, Masashi Yanagisawa, Yu Hayashi

**Affiliations:** ^1^International Institute for Integrative Sleep Medicine (WPI-IIIS), University of Tsukuba, Tsukuba, Japan; ^2^Doctoral Program in Biomedical Sciences, Graduate School of Comprehensive Human Sciences, University of Tsukuba, Tsukuba, Japan; ^3^Ph.D. Program in Human Biology, School of Integrative and Global Majors, University of Tsukuba, Tsukuba, Japan; ^4^Doctoral Program in Kansei, Behavioral and Brain Sciences, Graduate School of Comprehensive Human Sciences, University of Tsukuba, Tsukuba, Japan; ^5^Division of Molecular Brain Science, Research Institute of Traditional Asian Medicine, Kindai University, Osaka, Japan; ^6^Department of Molecular Genetics, University of Texas Southwestern Medical Center, Dallas, TX, United States; ^7^Life Science Center for Survival Dynamics, Tsukuba Advanced Research Alliance, University of Tsukuba, Tsukuba, Japan

**Keywords:** stress, sleep, depression, mouse, REM sleep

## Abstract

Repeated stress is a risk factor for mental disorders and can also lead to sleep disturbances. Although the effects of stress on sleep architecture have been investigated in rodents, the length of the stress exposure period in most studies has been limited to about 10 days, and few studies have analyzed the effects of chronic stress over a longer period. Here we investigated how sleep is affected in a mouse model of depression induced by 3 weeks of daily water immersion and restraint stress (WIRS). Sleep was recorded after 1, 2, and 3 weeks of stress exposure. Some stress-induced changes in several sleep measures were maintained across the 3 weeks, whereas other changes were most prominent during the 1st week. The total amount of non-rapid eye movement sleep (NREMS) was increased and the total amount of time spent awake was decreased across all 3 weeks. On the other hand, the amount of REMS during the dark phase was significantly increased in the 1st week compared with that at baseline or the 2nd and 3rd weeks. Electroencephalogram (EEG) power in the delta range was decreased during NREMS, although the total amount of NREMS was increased. These findings indicate that repeated WIRS, which eventually leads to a depression-like phenotype, differentially affects sleep between the early and subsequent periods. The increase in the amount of REMS during the dark phase in the 1st week significantly correlated with changes in body weight. Our results show how sleep changes throughout a long period of chronic stress in a mouse model of depression.

## Introduction

Sleep disturbances are major symptoms of various psychiatric disorders. More than 90% of patients with depression experience sleep disturbances (for review see Reynolds and Kupfer, 1987). Sleep architecture refers to the cycles of non-rapid eye movement sleep (NREMS) and REMS. Abnormalities in the sleep architecture, especially the REMS cycle, are frequently observed in depressed individuals (for reviews see Armitage, 2007; Nutt et al., 2008; Steiger and Kimura, 2010; Palagini et al., 2013; Medina et al., 2014). For example, several studies have demonstrated an increased density (Reynolds et al., 1985; Rotenberg et al., 2000; for reviews see Nutt et al., 2008; Steiger and Kimura, 2010; Palagini et al., 2013; Medina et al., 2014) and decreased latency of the first REMS episode (Reynolds et al., 1985; Papadimitriou et al., 1988; Hubain et al., 2006; for reviews see Armitage, 2007; Nutt et al., 2008; Steiger and Kimura, 2010; Palagini et al., 2013; Medina et al., 2014). A decrease in slow-wave sleep is also reported in patients with depression (Hubain et al., 2006; Lopes et al., 2007; for reviews see Armitage, 2007; Nutt et al., 2008; Steiger and Kimura, 2010; Palagini et al., 2013; Medina et al., 2014). To understand and overcome the sleep disturbances associated with psychiatric disorders such as depression, it is crucial to elucidate how changes in the sleep architecture emerge during the development of the disease.

Stress is a major environmental risk factor for psychiatric disorders. Especially, chronic stress and chronic exposure to high levels of stress hormones are associated with the development of depression in humans (for reviews see Lupien et al., 2009; Tafet and Nemeroff, 2016). Rodents are useful animal models for studying the effects of stress. Although careful interpretation is required, rodents exposed to chronic stress exhibit various phenotypes that resemble the features of depression, including despair-like and anhedonia-like behaviors (for reviews see Czéh et al., 2016; Slattery and Cryan, 2017; Wang et al., 2017). These phenotypes are alleviated by the administration of antidepressants that are effective in humans (for reviews see Willner, 2005; Mahar et al., 2014; Hare et al., 2017; Ramaker and Dulawa, 2017). Thus, the use of rodent models is expected to provide clues to the mechanisms underlying changes in the sleep architecture due to stress that potentially contribute to the development of psychiatric diseases. Various studies have examined the effects of stress on sleep in rodents. For example, exposure to acute restraint stress for 0.5–2 h increases the REMS (Rampin et al., 1991; Gonzalez et al., 1995; Bonnet et al., 1997; Marinesco et al., 1999; Meerlo et al., 2001; Koehl et al., 2002; Dewasmes et al., 2004; Paul et al., 2009; Rachalski et al., 2009). Social defeat stress has a similar effect (Meerlo and Turek, 2001; Henderson et al., 2017). REMS increases during the dark phase (Meerlo and Turek, 2001; Meerlo et al., 2001; Paul et al., 2009; Henderson et al., 2017) regardless of the timing of the exposure to stress (Koehl et al., 2002). This increase in REMS after daily stress is maintained throughout a 10-day period of chronic exposure (Henderson et al., 2017). One study, however, reported that the REMS amount increased significantly only after several days of daily stress exposure (Wells et al., 2017). In previous studies using restraint stress or social defeat stress, the exposure to stress was limited to a relatively short period ranging from a single day to approximately 10 days. Thus, the aim of this study was to analyze the effects of exposure to daily stress over a longer period.

In this study, we focused on water immersion and restraint stress (WIRS), which is a combination of restraint stress and water-immersion stress that leads to the robust emergence of depression-like behaviors (Miyata et al., 2011). Moreover, 3 weeks of WIRS alters white matter integrity in the corpus callosum, another feature in common with patients diagnosed with depression (Miyata et al., 2011, 2016). While social defeat stress was widely applied in previous studies, prolonged cycles could lead to injuries as well as damage to the electroencephalogram (EEG) and electromyogram (EMG) electrodes due to attacks by an aggressive mouse during the stress session. Thus, in the present study, we applied WIRS daily for 6 consecutive days per week for up to 3 weeks and analyzed the effect on the sleep architecture weekly.

## Materials and Methods

### Animals

All animal experiments were approved by the Institutional Animal Care and Use Committee of the University of Tsukuba, and all procedures were conducted in accordance with the Guidelines for Animal Experiments of the University of Tsukuba. Adult male C57BL/6J mice (8–19 weeks old) were used in this study. The mice were housed in a standard cage and maintained in controlled environment (23.5 ± 2.0°C, 51.0 ± 10.0% humidity, 12-h light/dark cycle). Food and water were available *ad libitum*.

### Surgery

The mice were anesthetized with isoflurane and placed in a stereotaxic frame (David Kopf Instruments, CA, United States). Core body temperature was maintained using a heating pad. EEG electrodes were stainless steel recording screws implanted epidurally over the parietal cortex (ML + 1.5 mm, AP + 1.0 mm from lambda) and the cerebellum (ML 0.0 mm, AP −6.5 mm from bregma). EMG electrodes were stainless steel Teflon-coated wires placed bilaterally into the trapezius muscles. The electrodes were fixed to the skull with dental cement (Super-Bond C&B set; Sun Medical, Shiga, Japan and Unifast II; GC Corporation, Tokyo, Japan). The mice were allowed to recover in their home cage for at least 3 weeks before transfer to the sleep recording chamber.

### EEG/EMG Recording and Analysis

Mice were attached to a recording cable and acclimatized to a sleep recording chamber for at least 3 days. EEG/EMG recordings were performed four times: baseline, 1st week (1W), 2nd week (2W), and 3rd week (3W) during the stress exposure period. For baseline sleep, the EEG/EMG was recorded for 24 h. For sleep following stress exposure, the EEG/EMG was recorded for 45 h after the stress session [Zeitgeber time (ZT) 3.0-] on Day 6 of each week. For the naïve mice, EEG/EMG recordings were performed two times: 3 and 6 weeks after surgery, corresponding to baseline and 3W of the stressed mouse group in terms of the period after surgery. The EEG/EMG data were filtered (band pass 0.5–64 Hz), and collected and digitized at a sampling rate of 128 Hz, and further filtered *post hoc* by software (EEG: high pass 0.5 Hz). EEG signals were subjected to fast Fourier transform and further analysis using SleepSign (Kissei Comtec, Nagano, Japan). The vigilance state in each epoch was manually classified as wake, NREMS, or REMS by every 4-s epoch based on EEG patterns of delta power (0.5–4.0 Hz), the theta power (6.0–10.0 Hz) to delta power ratio, and the integral of EMG signals. Epochs with high EMG and low delta power were classified as wakefulness. Epochs with high delta power and low EMG were classified as NREMS. Epochs with even lower EMG (suggestive of muscle atonia) and high theta power to delta power ratio were classified as REMS (Figure 2A). If a single epoch contained multiple states, the state with the longest duration was assigned. For EEG spectrum calculation, to avoid the effect of mixed states, any epochs which contained multiple states were excluded. For each individual, the average EEG power spectrum of each state was calculated and normalized using the average absolute value of the total EEG power across all frequencies and across all 24 h.

### Behavioral Analysis

All behavioral assays were performed during the light phase, between ZT 0.0 and 1.0. Prior to the behavioral experimental procedure, the mice were handled for 2 min twice daily for 5 days. The forced swim test (FST) and sucrose preference test (SPT) were performed on the 4th week, following 3 weeks of WIRS. Before each individual test session, the apparatus was sterilized with weak acidic water.

### Forced Swim Test

The FST was performed as described previously with some modifications (Hashikawa-Hobara et al., 2015). Briefly, mice were transferred to the behavioral testing room for acclimatization at least 10 min prior to the test session. The mice were placed individually in a plastic chamber (25 cm high and 20 cm in diameter) filled with water (23.5 ± 1.0°C) to a depth of 18 cm and forced to swim for 10 min. The light intensity was 60 lx. Mouse activity was recorded using a video camera and analyzed by visual observation. Immobility was defined as the state where motion seemed to stop or was minimized to only that required to keep the head above water. The data between 1 and 6 min from the start point were used for analysis. The analysis was conducted twice for each mouse, and the mean value was taken as the score of each mouse.

### Sucrose Preference Test

The SPT was performed as described previously with some modifications (Ramirez et al., 2015). Briefly, on Day 1, mice were each habituated in a standard cage with two bottles containing drinking water. On Day 2, one bottle of drinking water was replaced with 2% (w/v) sucrose (Nacalai Tesque, Inc., Kyoto, Japan) water. The bottles of drinking water and sucrose water were placed randomly in one of the two water bottle holders in the cage to prevent the mice from developing a side preference. The weight of the bottles was measured at the starting time and 24 h later. The positions of the two bottles were changed at 12 h to avoid the development of a side preference. Sucrose preference was calculated by the following index: (weight of consumed sucrose water)/[weight of consumed (sucrose water + drinking water)] × 100.

### Water Immersion and Restraint Stress

Water immersion and restraint stress was applied as described previously with some modifications (Mizoguchi et al., 2000; Miyata et al., 2011). Briefly, mice were restrained by a 50-ml conical polypropylene centrifuge tube containing multiple air holes. To prevent the mice from escaping, each tube was placed in a stretched net, and the net and tail were fixed in place with a rubber band. The tubes were immersed in water vertically to the level of the xiphoid process for 2 h between ZT 0.0 and ZT 2.5. Water temperature was adjusted to 22.0 ± 1.0°C. Mice were subjected to this stress session once a day for 6 consecutive days per week for 3 weeks. Body weight was measured before the stress session each day. For control mice, instead of exposure to 2 h of WIRS, the mice were placed in a novel cage for 2 h with no food or water. In an independent group of mice, core body temperature was measured using a heating pad system (Muromachi Kikai, Tokyo, Japan) before the stress session, immediately after (0), 0.5, 1, 1.5, or 2 h after the stress session on Day 1 and Day 5. While daily WIRS for 6–7 h induces gastric ulcers (Konturek et al., 1991; Brzozowski et al., 1993), we previously confirmed that the milder protocol used in the present study does not cause gastric ulcers (Mizoguchi et al., 2000; Miyata et al., 2011).

### Experimental Protocols

**FIGURE 1:**
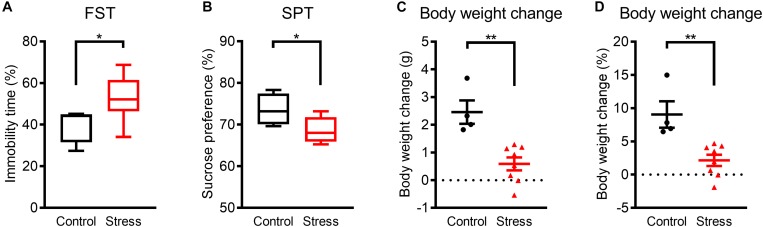
Mice exhibited depression-like phenotypes within 3 weeks of WIRS. **(A)** Immobility time in the FST. **(B)** Sucrose preference in the SPT. **(C,D)** Actual **(C)** and relative **(D)** body weight change. Control group:*N* = 4, stressed group: *N* = 8. **(A,B)** ^∗^*p* < 0.05, Mann–Whitney *U*-test; **(C,D)** ^∗∗^*p* < 0.01, Unpaired *t*-test. **(A,B)** Boxplots show 25% quartile, median, and 75% quartile of distributions; **(C,D)** Data are presented as means ± SEM.

**FIGURE 2:**
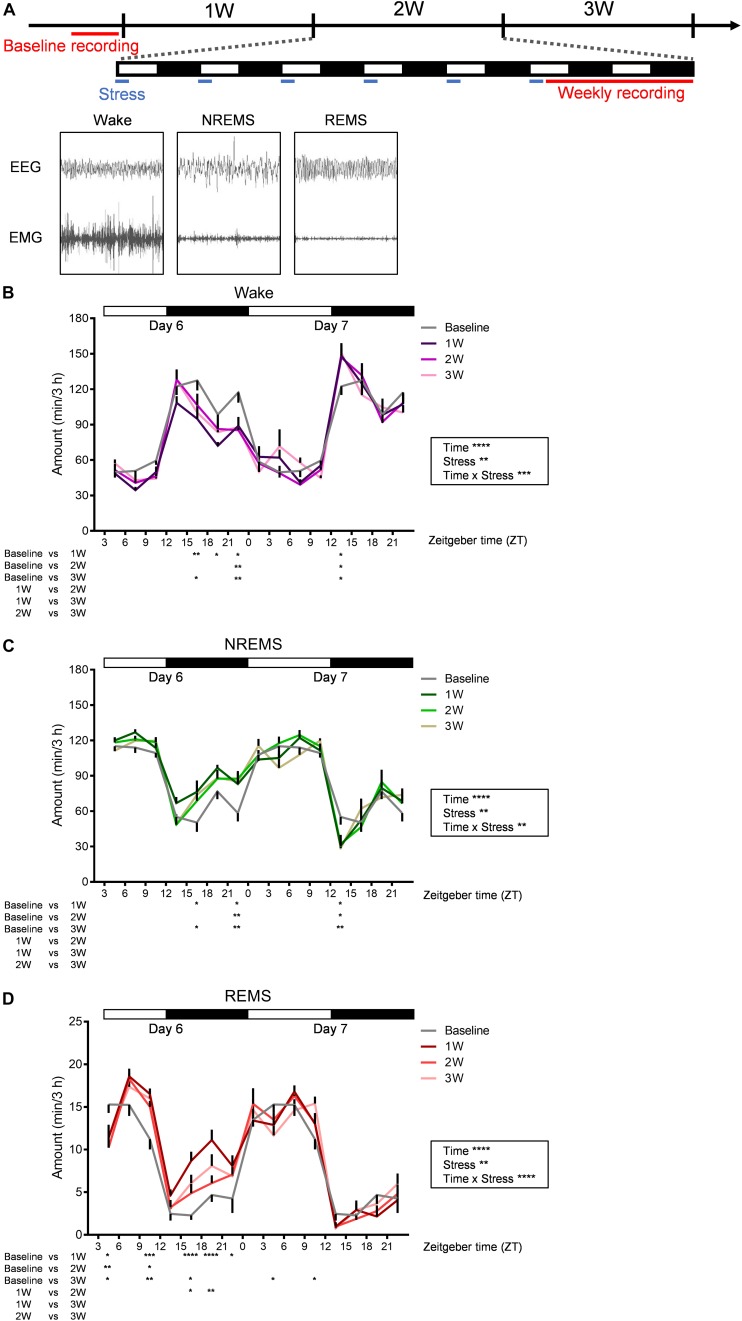
Diurnal sleep–wake cycles were affected by WIRS. **(A)** Experimental timeline and example of EEG (above) and EMG (bottom) patterns in each stage of wake (left), NREMS (middle), and REMS (right). **(B–D)** Daily variation in wake **(B)**, NREMS **(C)**, and REMS **(D)** every 3 h. Because the baseline recording was only 24 h, the same data were used as the control for the first and second day recordings at 1W, 2W, and 3W. *N* = 7. ^∗^*p* < 0.05, ^∗∗^*p* < 0.01, ^∗∗∗^*p* < 0.001, ^∗∗∗∗^*p* < 0.0001, two-way repeated measures ANOVA followed by Bonferroni’s multiple comparisons test. Two-way repeated measures ANOVA was applied over the entire 45 h recording period. Data are presented as means ± SEM.

**FIGURE 3:**
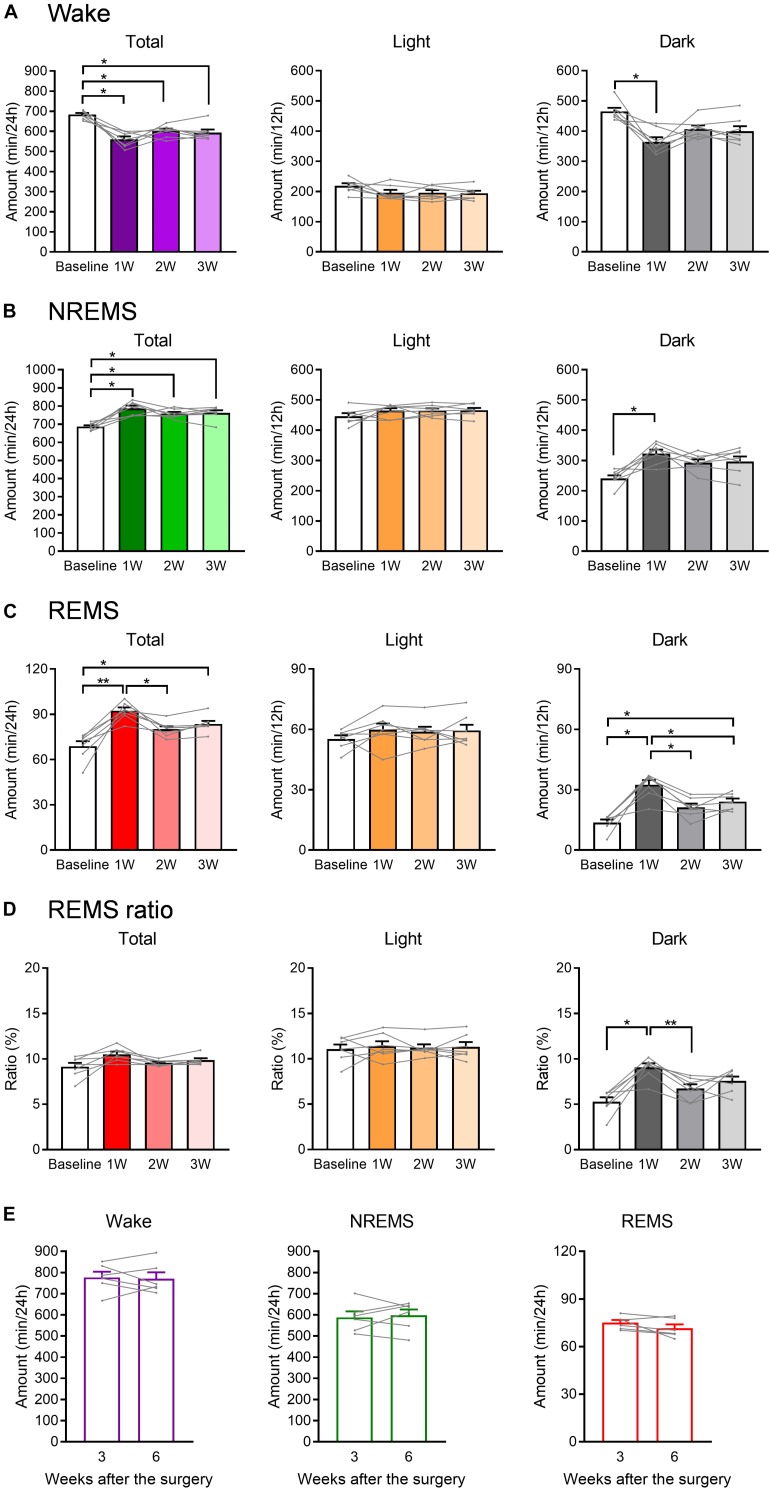
The total amount of each sleep/wake stage was affected by WIRS. **(A–C)** Total amount of wake **(A)**, NREMS **(B)**, and REMS **(C)** (24 h, light phase, dark phase). **(D)** Ratio of REMS amount to the total sleep amount (24 h, light phase, dark phase). **(E)** Total amount of each sleep/wake stage in naïve mice 3 and 6 weeks after surgery. **(A–D)** *N* = 7, **(E)** *N* = 6. **(A–D)** ^∗^*p* < 0.05, ^∗∗^*p* < 0.01, one-way repeated measures ANOVA followed by Bonferroni’s multiple comparisons test. **(E)** Paired *t*-test. **(A–D)** Data are a summary of the first 24 h of the 45 h recording periods and are presented as means ± SEM.

**FIGURE 4:**
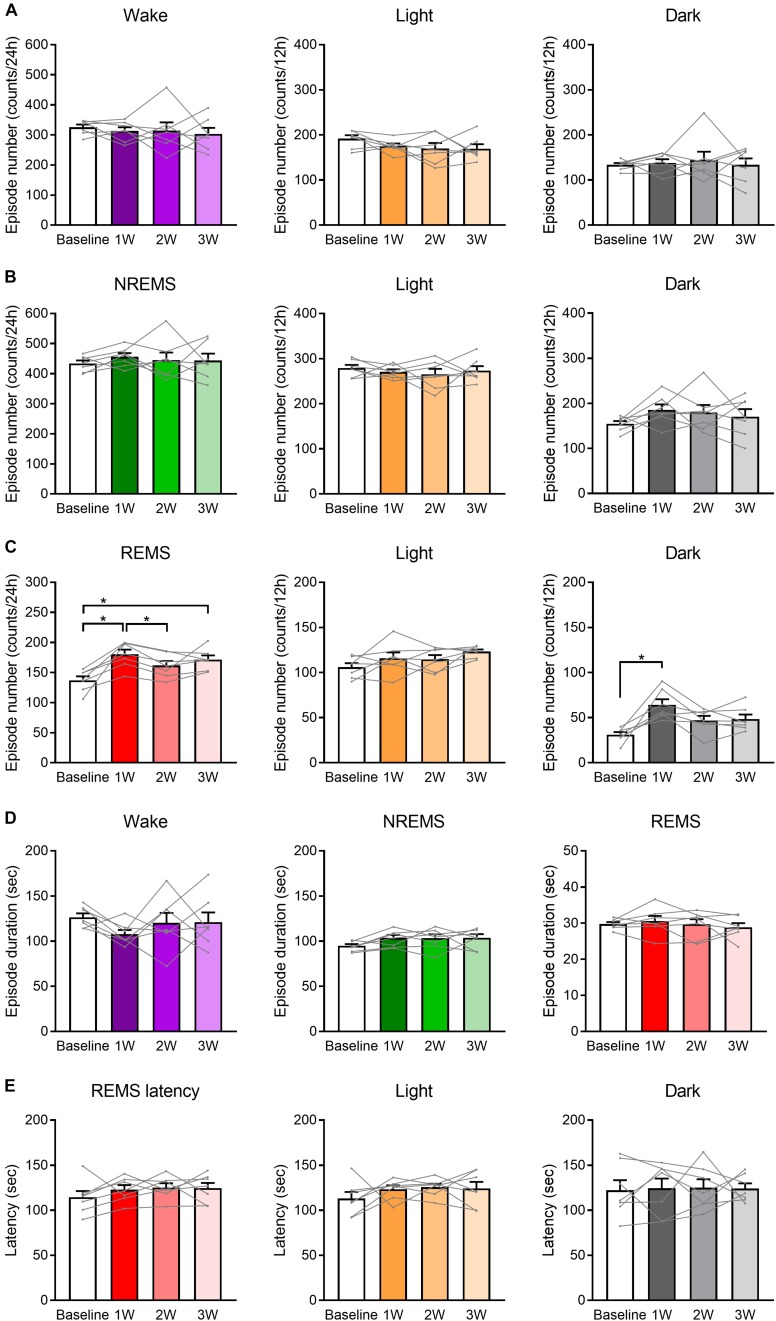
Number of REMS episodes was increased by WIRS. **(A–C)** Number of episodes of wake **(A)**, NREMS **(B)**, and REMS **(C)** (24 h, light phase, dark phase). **(D)** Episode duration in each stage of wake, NREMS, and REMS (average of 24 h). **(E)** Mean REMS latency (24 h, light phase, dark phase). *N* = 7. **(A–E)** One-way repeated measures ANOVA followed by Bonferroni’s multiple comparisons test. Data are a summary of the first 24 h of the 45 h recording periods and are presented as means ± SEM. ^∗^*p* < 0.05.

**FIGURE 5:**
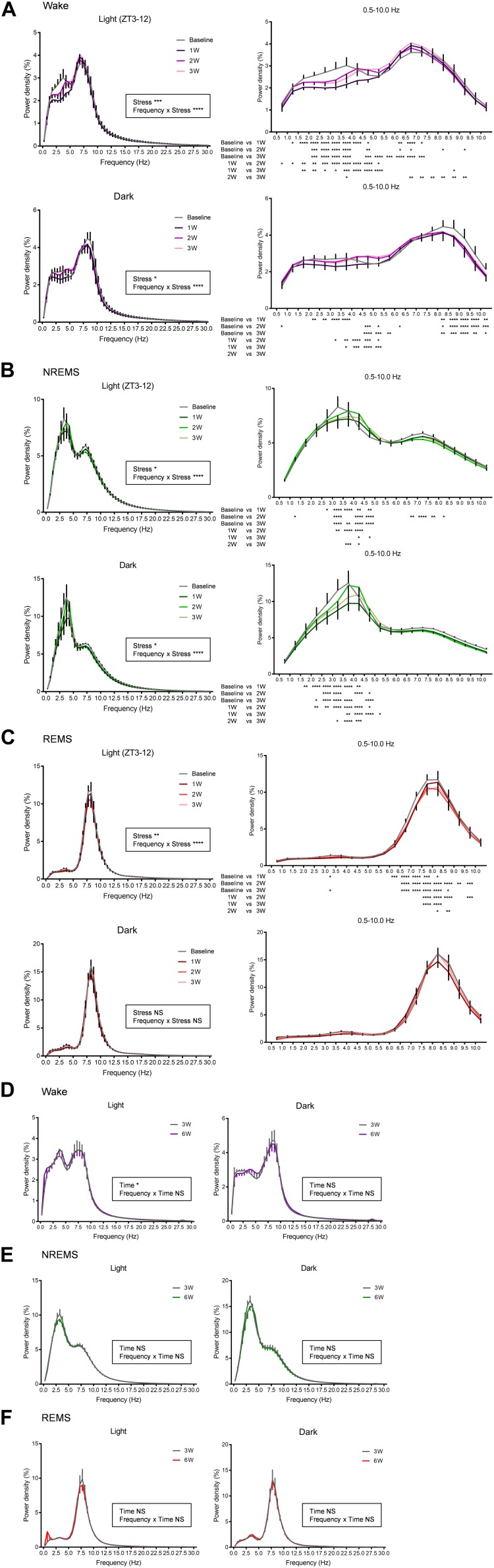
EEG power spectra were affected by WIRS. **(A–C)** EEG power spectra during wake, NREMS, and REMS (the first light phase and dark phase following WIRS). **(D–F)** EEG power spectra during wake, NREMS, and REMS (light phase, dark phase) 3 and 6 weeks after surgery in naïve mice. **(A–C)** *N* = 5 (two of seven mice were excluded due to contamination of large noise during wake), **(D–F)** *N* = 5. ^∗^*p* < 0.05, ^∗∗^*p* < 0.01, ^∗∗∗^*p* < 0.001, ^∗∗∗∗^*p* < 0.0001, two-way repeated measures ANOVA followed by Bonferroni’s multiple comparisons test. Two-way repeated measures ANOVA was applied to the entire 0.5–30 Hz data. The *post hoc* test results are shown only for the 0.5–10 Hz region as there were no significant points outside of this region. Data are presented as means ± SEM.

**FIGURE 6:**
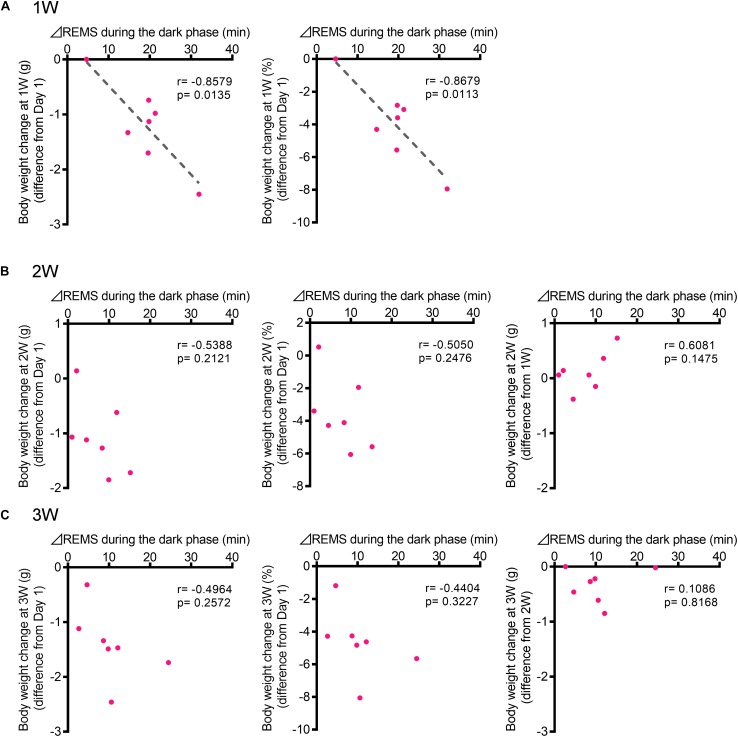
The increase in the REMS amount during the dark phase correlated with the body weight change at 1W. **(A)** Correlation between change in REMS amount compared with that at baseline during the dark phase at 1W **(A)**, 2W **(B)**, and 3W **(C)** [**Left:** actual body weight change (difference from Day 1), **Middle:** normalized as a percentage (difference from Day 1), and **Right:** actual body weight change (difference from previous week)]. Change in REMS amount was calculated as follows: (the amount of REMS during the 12 h of dark phase after WIRS at each week) – (the amount of REMS during the 12 h of dark phase in the baseline recording). *N* = 7. **(A–C)** Pearson correlation coefficients.

**FIGURE 7:**
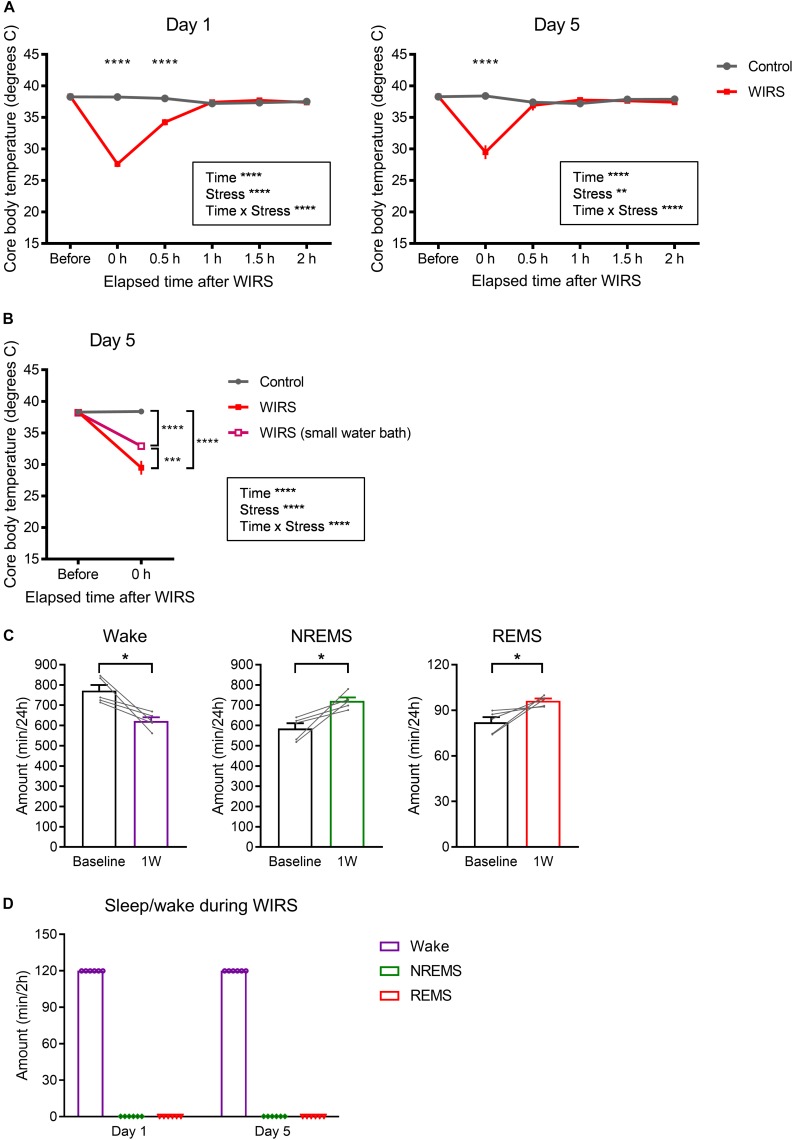
Effects of WIRS on core body temperature and assessment of sleep/wake during the WIRS. **(A)** Changes in core body temperature before and after the WIRS session on Day 1 and Day 5 during 5 consecutive days of WIRS. **(B)** Comparison of core body temperature before and after the WIRS session on Day 5 between usage of two types of water baths (one was the original water bath and the other was a smaller water bath). **(C)** Total amount of each sleep/wake stage in the 24 h following 6 consecutive days of WIRS with the smaller water bath. **(D)** Total amount of each sleep/wake stage during the WIRS session. **(A,B)** Control: *N* = 4, WIRS: *N* = 4, WIRS using smaller water bath: *N* = 6. **(C)** *N* = 5. **(D)** *N* = 6. **(A,B)** ^∗∗^*p* < 0.01, ^∗∗∗^*p* < 0.001, ^∗∗∗∗^*p* < 0.0001, two-way repeated measures ANOVA followed by Bonferroni’s multiple comparisons test. **(C)** ^∗^*p* < 0.05, paired *t*-test.

The number of animals that were used for each study is as follows. For behavioral analyses shown in Figures 1A–D, control group: *N* = 4, stressed group: *N* = 8. For EEG/EMG recordings shown in Figures 2B–D, 3A–D, 4A–E, 6A–C: *N* = 7; Figures 3E, 7D: *N* = 6; and Figures 5A–F, 7C: *N* = 5. For core body temperature measurement shown in Figures 7A,B, control group: *N* = 4, WIRS group: *N* = 4, WIRS with small water bath: *N* = 6. Throughout the 3 weeks of experiment, mice were individually housed in the sleep recording cages except during the WIRS.

### Statistics

All data were analyzed using Prism 7 (Graph Pad, San Diego, CA, United States) or Excel (Microsoft, Redmond, WA, United States). For behavioral analyses, a Mann–Whitney *U*-test was performed. Changes in body weight were analyzed using an unpaired *t*-test. For analysis of daily variations of wake, NREMS, and REMS, a two-way repeated measures ANOVA was performed followed by Bonferroni’s multiple comparisons test. Time spent in each stage of wake, NREMS, and REMS, and the ratio of REMS to the total sleep amount was analyzed using a one-way repeated measures ANOVA followed by Bonferroni’s multiple comparisons test. The effects of water bath type or period from surgery on time spent in each stage of wake, NREMS, and REMS were analyzed using paired *t*-test. Both number of episodes and mean episode duration in each stage of wake, NREMS, and REMS, and REMS latency were also analyzed using a one-way repeated measures ANOVA followed by Bonferroni’s multiple comparisons test. The EEG power spectra were analyzed using a two-way repeated measures ANOVA followed by Bonferroni’s multiple comparisons test. For analysis of the correlation between the REMS increase during the dark phase and body weight change, Pearson correlation coefficients were evaluated. Changes in core body temperature were analyzed using a two-way repeated measures ANOVA followed by Bonferroni’s multiple comparisons test. Where applicable, all tests were two-tailed.

## Results

### Assessment of Depression-Like Phenotypes Following WIRS

Prior to analyzing the effects of WIRS on sleep architecture, we confirmed the emergence of a depression-like phenotype by exposure to the stress for 3 weeks. In assessing depression-like behavior, the FST was applied as a measure for despair-like phenotypes and the SPT was applied as a measure for anhedonia-like phenotypes. Stressed mice showed an increase in immobility time in the FST (Figure 1A) and a decrease in sucrose preference in the SPT (Figure 1B) compared with control mice. In addition, body weight gain during the 3 weeks was lower in stressed mice than in control mice (Figures 1C,D). These results are consistent with previous reports on depression-like phenotypes caused by WIRS (Miyata et al., 2011; Hashikawa-Hobara et al., 2015; Dai et al., 2018).

### Effect of WIRS on the Sleep/Wake Cycle

We next investigated the effects of 3 weeks of WIRS on sleep/wake (Figure 2). Figure 2A shows the experimental timeline of the stress exposure and EEG/EMG recordings. EEG/EMG was recorded each week for 45 h beginning on Day 6 (ZT3.0-). In the baseline sleep recording, EEG/EMG was recorded for 24 h.

Stress induced various changes in the amount of sleep/wake. At some times of day, sleep/wake was similarly affected at 1W, 2W, and 3W. By contrast, at some other times of day, sleep/wake was affected most strongly at 1W whereas the effect was milder at 2W and 3W.

Across all weeks during the stress session, at ZT 21.0–24.0 on Day 6 (∼18 h after the stress), there was a decrease in wake and concomitant increase in NREMS compared with that at baseline (Figures 2B,C). In contrast, across all weeks during the stress session, ZT 12.0–15.0 at Day 7 (∼33 h after the stress), there was an increase in wake and concomitant decrease in NREMS (Figures 2B,C).

Although the changes in the amount of wake and NREMS appeared to mirror each other, the change in the amount of REMS differed from that in the other stages. Across all weeks, at ZT 3.0–6.0 on Day 6 (immediately after the stress), REMS was decreased, and at ZT 9.0–12.0 on Day 6 (6 h after the stress), REMS was increased, while the amounts of wake and NREMS were not significantly affected in either period (Figure 2D). Another feature of REMS was that the effect of stress changed dynamically across the whole stress exposure period. This was most obvious during the dark phase of Day 6, especially at ZT 15.0–21.0, where the REMS amount was largely increased at 1W, whereas the increase appeared more blunted at 2W and 3W (Figure 2D).

### Effect of WIRS on Various Sleep/Wake Parameters

We next compared the total amount in each stage of wake, NREMS, and REMS during the light phase, dark phase, or 24 h after the stress exposure (Figure 3). Again, the changes in wake and NREMS appeared to mirror each other (Figures 3A,B). Total amount of NREMS in the 24 h after the stress exposure was increased at all weeks compared with that at baseline, whereas the amount of wake was decreased. For REMS, the total amount at 24 h was largely increased at 1W, but not at 2W (Figure 3C).

When 24 h were further divided into the light and dark phases, no significant change in the amount of sleep or wake was detected in the light phase. In contrast, in the dark phase, the total amounts of both NREM and REMS were increased at 1W. Importantly, the ratio of REMS to total sleep was also increased during the dark phase at 1W, suggesting that the increase in REMS was not simply a result of an overall increase in sleep (Figure 3D).

To confirm that these changes in sleep parameters were not due simply to the gradual deterioration of implanted EEG/EMG electrodes, EEG/EMG recordings were also performed in naïve mice at 3 and 6 weeks after surgery, corresponding to baseline and 3W of the stressed mice in terms of the period after surgery. As a result, the total amount in each stage of wake, NREMS, and REMS did not significantly change in these mice across the period (Figure 3E).

Analyses of the number and duration of each episode of sleep/wake (Figure 4) revealed that the increase in the REMS amount could be largely attributed to the increase in the number, not duration, of REMS episodes (Figures 4C,D). For NREMS and wake, we detected no significant change in either the episode number or duration, and thus we could not determine which factor contributed more to the change in the total amount of each stage.

Additionally, we analyzed the latency to REMS, which is defined as the duration of the NREMS episode immediately preceding the first REMS episode. We detected no significant effect of the stress on REMS latency (Figure 4E).

### Effect of WIRS on the EEG Power Spectrum

We next analyzed the effect of stress exposure on the EEG power spectrum (Figure 5). For the wake stage, during the light phase, there was a decrease in delta power and an increase in theta power across all weeks, perhaps reflecting a more attentive state (Figure 5A). By contrast, during the dark phase, theta power was decreased across all weeks. In addition, delta power was decreased at 1W.

For the NREMS stage, both during the light phase and dark phase, a decrease in power in the delta range (0.5–4.0 Hz) was detected across all weeks compared with that at baseline (Figure 5B). This effect was strongest at 1W.

For the REMS phase, in contrast to the change in total amount detected during the dark phase, the change in the EEG power spectrum was detected at the light phase (Figure 5C). During the light phase, theta power was decreased across all weeks compared with that at baseline. Notably, the reduction in theta power was more pronounced at 2W and 3W compared with that at 1W. This is in large contrast to most other changes in the sleep/wake, which were either most drastic at 1W or comparable across all weeks. During the dark phase, in contrast to the dynamic change in the amount of REMS (Figure 3C), no significant change in the EEG spectrum was detected (Figure 5C).

We confirmed that the EEG power spectrum did not significantly differ in the naïve mice between 3 and 6 weeks after surgery (Figures 5D–F).

### Correlation Between Change in REMS Amount During the Dark Phase and Body Weight Change

To investigate whether changes in sleep are correlated with the development of depression-like phenotypes, we next focused on body weight. A strong effect of stress exposure on sleep/wake was observed in the amount of REMS during the dark phase (Figures 2, 3). Thus, we tested whether the change in the REMS amount during the dark phase was correlated with the change in body weight. At 1W, there was a significant negative correlation (Figure 6A). By contrast, there was no significant correlation at 2W or 3W (Figures 6B,C).

### Effects of WIRS on Core Body Temperature

Besides stress, WIRS might have other effects that also affect subsequent sleep. Here, we assessed the effect on core body temperature. Immediately after the WIRS session, core body temperature significantly declined, which recovered to baseline level within 1 h (Figure 7A). Applying WIRS using a smaller water container partly suppressed the core body temperature decline (Figure 7B). Nonetheless, 6 consecutive days of WIRS under this condition resulted in a high amount of subsequent REM sleep comparable to the original WIRS condition (Figure 7C).

### Assessment of Sleep/Wake During WIRS

We tested whether mice slept during the WIRS. EEG/EMG recording from mouse undergoing WIRS indicated that mice were completely awake throughout the WIRS (Figure 7D).

## Discussion

To our knowledge, this is the first rodent study to examine the effect of chronic stress on the sleep architecture by applying WIRS for as long as 3 weeks. While many rodent studies have applied social defeat stress to address the effect of stress on sleep, the longest period tested was 10 days because social stress may cause severe injuries to the animal and damage the EEG/EMG electrodes. The WIRS protocol is advantageous for circumventing such potential problems.

Comparisons of the sleep architecture between the 1W, 2W, and 3W, and baseline conditions revealed that stress differentially affected sleep at 1W compared with that at 2W and 3W. For example, while the stress induced an overall increase in REMS, this effect was especially strong at 1W. The correlation of the change in body weight with the change in the amount of REMS at 1W further supports the strong association between stress and sleep, especially during the early period. It is possible that, after 1W, animals gradually adapted to the repeated WIRS sessions and experienced less stress. The fact that the body weight continued to decrease to a similar extent at 2W and 3W as at 1W, however, does not support this possibility. It is also possible that prolonged exposure to a stressful environment attenuates the adaptive changes in sleep in response to a stressful environment. Further studies should be performed to examine whether the observed attenuation in the sleep alterations at later periods contributes to the emergence of depression-like behaviors.

Compared with wake and NREMS, changes in REMS occurred more rapidly. In the light phase immediately following stress exposure, REMS was initially strongly decreased and subsequently increased, and these responses occurred at a timing when no significant change was observed in the amount of NREMS or wake. The initial suppression of REMS might be a result of increased corticosterone, as a systemic increase in cortisol is suggested to inhibit REMS (Born et al., 1991). The subsequent strong increase in REMS might in part be a result of a homeostatic response to the prior reduction of REMS, although other mechanisms could also be involved. In the following dark phase, both REMS and NREMS amounts were increased, but the increased REMS amount was more pronounced as the ratio of REMS to the total sleep amount was also increased. This increase in the amount of REMS and NREMS during the dark phase was attenuated at 2W and 3W. The overall increase in the REMS amount is consistent with findings in mice exposed to 10 days of social defeat stress (Wells et al., 2017). Moreover, an increase in the REMS amount is reported in patients with depression (Papadimitriou et al., 1988; for reviews see Armitage, 2007; Palagini et al., 2013). The increased REMS amount might be explained by the REMS-promoting effect of corticotropin-releasing hormone in the forebrain (Kimura et al., 2010). We must note the possibility that effects other than stress that were caused by WIRS might have also contributed to changes in the sleep parameters. For example, effects on metabolism or thermoregulation might have contributed to changes in REMS (for review see Parmeggiani, 2003). In rodents, REMS amount changes dynamically according to environmental temperature, with a peak around 29–30°C (Szymusiak and Satinoff, 1981; Kumar et al., 2009; for review see Cerri et al., 2017). The initial suppression and subsequent increase of REMS amount observed in our study might be in part due to the rapid decline and subsequent recovery of core body temperature caused by WIRS. However, under conditions of WIRS in which the decline in core body temperature was partly suppressed, REMS amount was still significantly increased to a comparable level, suggesting that such changes in REMS was not solely due to the changes in the core body temperature. During the WIRS session, the mice were sleep-deprived, which might have also contributed to changes in subsequent sleep. However, effects such as the decrease in delta power during NREMS and increase in the ratio of REMS amount to total sleep were not observed after total sleep deprivation (Mochizuki et al., 2004).

Due to uncertainties about the function of REMS, it is difficult to speculate whether the increase in the REMS amount at 1W and its dampening in the subsequent weeks are beneficial or non-beneficial for an animal coping with stress. Antidepressants such as monoamine oxidase inhibitors and selective serotonin reuptake inhibitors reduce REMS (Mayers and Baldwin, 2005). Moreover, REMS deprivation has an antidepressant effect (Vogel et al., 1975). Thus, an increase in REMS might negatively affect recovery from psychiatric diseases. However, because antidepressants act on many brain regions and REMS deprivation is usually followed by a large REMS rebound, it is difficult to differentiate the direct effects of REMS reduction from these other effects. When sleep was recorded from subjects that underwent life-threatening experiences, subjects who had a longer duration of REMS episodes during the acute period had a lower tendency to develop subsequent posttraumatic stress disorder, perhaps supporting the notion that REMS plays a beneficial role (Mellman et al., 2002). Future studies using optogenetics or chemogenetics to manipulate REMS might provide more clues as to how REMS is involved in coping with stress.

Chronically stressed mice also exhibited changes in the EEG power spectrum. In NREMS, especially during the dark phase, the delta power was decreased across all weeks. This is consistent with the observations of decreased slow-wave sleep in patients with depression (Hubain et al., 2006; Lopes et al., 2007; for reviews see Armitage, 2007; Nutt et al., 2008; Steiger and Kimura, 2010; Palagini et al., 2013; Medina et al., 2014). Slow-wave sleep is characterized by a decrease in the blood cortisol levels (Gronfier et al., 1997). Moreover, slow-wave activity itself may contribute to reducing cortisol levels (Besedovsky et al., 2017). Thus, it is possible that the decrease in delta power contributes to increase the cortisol levels and the emergence of depression-like phenotypes. In REMS, theta power was decreased, especially during the light phase. In contrast to most other changes in sleep/wake, which were either strongest at 1W or comparable across all weeks, the reduction in theta power was more pronounced at 2W and 3W. In mice, the surface EEG theta power mainly originates from the hippocampus. Thus, a progressive reduction of the theta power might reflect some accumulating damage to the hippocampus. Shrinkage of the hippocampal volume is well known to occur in patients with depression (Schmaal et al., 2016), and chronic stress in mice may have a similar effect.

While the increase in the REMS amount observed in the present study in mice is consistent with findings in human patients with depression (Papadimitriou et al., 1988; for reviews see Armitage, 2007; Palagini et al., 2013), some other characteristics of sleep in depressed patients, including shortening of the REMS latency (Reynolds et al., 1985; Papadimitriou et al., 1988; Hubain et al., 2006; for reviews see Armitage, 2007; Nutt et al., 2008; Steiger and Kimura, 2010; Palagini et al., 2013; Medina et al., 2014) and sleep fragmentation (Rotenberg et al., 2000; Lopes et al., 2007; for reviews see Nutt et al., 2008; Steiger and Kimura, 2010; Palagini et al., 2013; Medina et al., 2014) were not observed in the present study. This might be a limitation of using WIRS to model human stress disorders and depression. In mice, 10 days of social defeat stress results in shortened REMS latency (Wells et al., 2017) and long-term exposure to various types of unpredictable mild stressors results in fragmented sleep (Nollet et al., 2019). Thus, it appears that each model has advantages for recapitulating human diseases. Some studies, however, report no significant difference in the REMS latency in patients with depression (Rotenberg et al., 2000; Lopes et al., 2007). Thus, it is possible that depression itself is a disorder with a spectrum of diverse sleep characteristics.

## Conclusion

In summary, this study shows how 3 weeks of WIRS, which is a robust protocol for inducing depression-like behaviors in mice, affects sleep architecture. Several changes observed in the present study recapitulated the sleep disturbances that occur in patients with depression. Moreover, the results demonstrated that mouse sleep exhibits a graded change in response to stress applied for 3 weeks. The findings of this study provide a platform for future studies to address how sleep is affected by chronic stress and how the changes in sleep induce other behavioral or physiologic alterations.

## Data Availability Statement

Data sets are available from the corresponding author upon reasonable request.

## Ethics Statement

The animal study was reviewed and approved by the Institutional Animal Care and Use Committee of the University of Tsukuba.

## Author Contributions

SY, C-YL, and YH conceived and designed the experiments with advice from SM. SY, C-YL, and MKas performed the experiments with advice from MKan, TH, and SM. SY and YH analyzed the data and wrote the manuscript with advice from SM and MY.

## Conflict of Interest

The authors declare that the research was conducted in the absence of any commercial or financial relationships that could be construed as a potential conflict of interest.
